# Protective effects of carnosol against oxidative stress induced brain damage by chronic stress in rats

**DOI:** 10.1186/s12906-017-1753-9

**Published:** 2017-05-04

**Authors:** Saeed Samarghandian, Mohsen Azimi-Nezhad, Abasalt Borji, Mohammad Samini, Tahereh Farkhondeh

**Affiliations:** 1 Department of Basic Medical Sciences, Neyshabur University of Medical Sciences, Neyshabur, Iran; 2 Department of Medical Genetics, School of Medicine, Mashhad University of Medical Sciences, Mashhad, Iran; 3 Department of Neurosurgery, Faculty of Medicine, Mashhad University of Medical Sciences, Mashhad, Iran; 4 Immunogenetic and Cell Culture Department, Immunology Research Center, School of Medicine, Mashhad University of Medical Sciences, Mashhad, Iran

**Keywords:** Carnosol, Brain, Behavior, Restrain stress, Oxidative stress

## Abstract

**Background:**

Oxidative stress through chronic stress destroys the brain function. There are many documents have shown that carnosol may have a therapeutic effect versus free radical induced diseases. The current research focused the protective effect of carnosol against the brain injury induced by the restraint stress.

**Methods:**

The restraint stress induced by keeping animals in restrainers for 21 consecutive days. Thereafter, the rats were injected carnosol or vehicle for 21 consecutive days. At the end of experiment, all the rats were subjected to his open field test and forced swimming test. Afterwards, the rats were sacrificed for measuring their oxidative stress parameters. To measure the modifications in the biochemical aspects after the experiment, the activities of malondialdehyde (MDA), reduced glutathione (GSH), as well as superoxide dismutase (SOD), glutathione peroxidase (GPx), glutathione reductase (GR) and catalase (CAT) were evaluated in the whole brain.

**Results:**

Our data showed that the animals received chronic stress had a raised immobility time versus the non-stressed animals (*p* < 0.01). Furthermore, chronic stress diminished the number of crossing in the animals that were subjected to the chronic stress versus the non-stressed rats (*p* < 0.01). Carnosol ameliorated this alteration versus the non-treated rats (*p* < 0.05). In the vehicle treated rats that submitted to the stress, the level of MDA levels was significantly increased (*P* < 0.001), and the levels of GSH and antioxidant enzymes were significantly decreased versus the non-stressed animals (*P* < 0.001). Carnosol treatment reduced the modifications in the stressed animals as compared with the control groups (*P* < 0.001). All of these carnosol effects were nearly similar to those observed with fluoxetine.

**Conclusion:**

The current research shows that the protective effects of carnosol may be accompanied with enhanced antioxidant defenses and decreased oxidative injury.

## Background

Stress is an uncontrolled psychological or emotional condition that disturbs cellular homeostasis [1]. Among the many types of tissues in the body, the brain is more susceptible to stress because of its high levels of intracellular mediators that are involved in the stress response (glucocorticoid receptors) [2]. Stress may induce a variety of central nervous system abnormalities, including depression, anxiety, locomotor activity, and cognitive function [3]. The main mechanism(s) associated with stress-induced behavioral disorders is the activation of the hypothalamo-hypophyseal-adrenal (HPA) axis, which involves elevation of the brain corticosterone level [4]. The increased level of corticosterone results in a rapid depolarization that evoked glutamate release in the cortical and limbic regions [5]. The over-production of glutamate can lead to a mitochondrial dysfunction and an increase in metabolic rate [6]. The increased metabolic rate produces extra free radicals, leading to an imbalance between reactive oxygen species (ROS) generation and anti-oxidant system [7]. These free radical species result in oxidative damage to different molecules in cells, such as proteins, lipids, and nucleic acids [7]. Brain is especially sensitive to oxidative stress due to its high content of readily oxidizable fatty acids, high consumption of oxygen, and low levels of antioxidants. The increased ROS and also lowered levels of antioxidants have been associated with the pathogenesis of neurodegenerative diseases such as depression and cognitive impairment [8]. However, the enhancement of antioxidant system may be effective to combat against the excessive ROS production [9–11]. According to strong evidences, the several natural antioxidants may be effective against stress-induced mental health complications [12]. Carnosol (CS) is a natural polyphenol (dietary diterpene) seen in plants belonging to Lamiaceae family (Mint Family) such as sage, rosemary, lavenders, and oregano [13]. Studies indicated that CS has many pharmacological effects, including antioxidant, anti-inflammatory, anti-microbial, and anti-cancer activities [14, 15]. The antioxidant activity of CS is activated via the catechol hydroxyl and is changed to a carnosol quinine. This quinone derivative is the main antioxidant products of CS, and has very weak antioxidant activity, but, under proper conditions the antioxidant activity can be ameliorated [14]. Previous studies indicated that culinary herbs such as rosemary may be effective for treating the neurodegenerative disorders in animal models. Recently, it was observed that the hydroalcoholic crude extract of *R. officinalis* as well as essential oil, the isolated compounds CS and betulinic acid caused an antidepressant-like effect in mice by evaluating the forced swimming test (FST) and tail suspension test (TST), that are the predictive tests of antidepressant activity, through a mechanism dependent on the interaction with the monoaminergic systems [16]. However, the involvement of this effect on brain and its anti−/pro-oxidative aspect has not yet been studied. Considering the protective effect of CS on the function of free radicals, the study was designed to investigate the protective effect of CS on the behavioral changes in the FST, open field test (OFT) and hippocampal antioxidant imbalance induced by the chronic restraint stress. We chose the antidepressant, fluoxetine, as a positive control in our experiment.

## Methods

### Reagents

All chemicals were purchased from Sigma-Aldrich Chemical (St. Louis, USA). The corticosterone ELISA kit was purchased from Cusabio (Cusabio Biotech Co., Ltd).

### Animals

Wistar albino rats (213 ± 10.2 g) were bred at the university experimental animal care center. Animals were maintained under standard environmental conditions and had free access to standard rodent feed and water.

### Study design

Rats were randomly divided into the nine experimental groups (8 rats per group) as follows: 1) Vehicle (Veh) + no-stress (NS) (Veh-NS); 2) Vehicle + stress (Veh-S), 3) CS (1 mg/kg, IP) + no-stress (CS1-NS), 4) CS (5 mg/kg, IP) + no-stress (CS5-NS); 5) CS (10 mg/kg, IP) + no-stress (CS10-NS), 6) CS (1 mg/kg, IP) + stress (CS1-S), 7) CS (5 mg/kg, IP) + stress (CS5-S), 8) CS (10 mg/kg, IP) + stress (CS10-S), 9) Fluoxetine (Flu) (10 ml/kg, IP) + stress (Flu-S). Protocols were approved by the Ethical Committee (The Ethical Research Committee of Mashhad University of Medical Sciences). The restraint stress was performed using a rodent restrainer made of plexiglass that closely fit the rats’ body. For the chronic restraint stress, the rats were kept in the restrainers for 1 h per day for 21 consecutive days. The animals received systemic administrations of the vehicle (3% dimethyl sulfoxide-DMSO) or CS daily for 21 days [17]. After the experimental period, all the rats were released from their enclosure and 40 min post-release were submitted to the FST, OFT and finally were sacrificed for evaluation the biochemical parameters.

### Behavioral evaluations

#### Forced swimming test (FST)

Briefly, the rats were individually forced to swim in an open cylindrical container (diameter 10 cm, height 25 cm), containing 19 cm of water (depth) at 25 ± 1 °C; the total duration of immobility was measured during a 6-min test period by observers blind to the treatment conditions. Each rat was judged to be immobile when it ceased struggling and remained floating motionless in the water, making only those movements necessary to keep its head above water [18].

#### Open field test (OFT)

To assess possible interferences on locomotor activity, the rats were evaluated in the open field paradigm as previously described [19, 20]. Rats were individually placed in a wooden box (40 × 60 × 50 cm) with the floor divided into 12 equal squares and the number of crossings with all paws was manually counted in a 6 min period. The light was maintained at the minimum to avoid anxiety behavior and the apparatus was cleaned with a solution of 10% ethanol between tests in order to hide animal clues [19, 20].

### Biochemical analysis

#### Tissue preparation

For biochemical tests, the animals were anesthetized with ether and blood was subsequently collected from the retro-orbital sinus. Blood and sera were separated by centrifugation at 5000 RPM for 5 min for corticosterone measurement. Then, the hippocampi were removed and homogenized in 50 mM phosphate buffer, pH 7.4 and centrifuged at 16,000×g, at 4 °C for 20 min. The homogenate and supernatant were used for the assays.

### Corticosterone evaluation

Under deep anesthesia, blood was collected from the retro-orbital sinus of the rats. Blood was allowed to clot and the sera was separated using centrifugation at 5000 RPM for 5 min and stored at −80 °C until use. The total serum level of corticosterone was measured by ELISA kits (CORT ELISA Kit CSB-E07014r).

### Measurement of MDA

Malondialdehyde (MDA) results from degradation of polyunsaturated lipids. The production of this substance is used as a biomarker to measure the level of lipid peroxidation. MDA reacts with thiobarbituric acid (TBA) as a thiobarbituric acid reactive substance (TBARS) to form a 1:2 MDA-TBA adduct, which absorbs at 532 nm. Thus, the quantity of TBARS is proportionate to the amount of MDA. The concentration of TBARS is determined according to a method of Mihara and Uchiyama. The concentration of TBARS was calculated using the MDA standard curve and was expressed as nmol/mg of protein [20].

### Estimation of GSH

The GSH level was measured by the method of Beutler et al. (1963) [21]. Briefly, 0.1 ml of sample was added to 0.9 ml distilled water and 1.5 ml of precipitating reagent (3.34 g meta-phosphoric acid, 0.4 g EDTA and 60.0 g sodium chloride). Tubes were shaken and allowed to stand for 5 min at the room temperature (25 ± 1 °C). The mixture was centrifuged for 15 min at 4000 RPM at 4 °C. In the 1.0 ml supernatant, 4.0 ml of phosphate solution (0.3 M disodium hydrogen phosphate) and 0.5 ml 5–50-dithiobis-(2-nitrobenzoic acid) (DTNB) (80 mg in 1% sodium citrate) were added. The development of yellow color complex was read immediately at 412 nm on a spectrophotometer. A standard curve using the GSH level was prepared and GSH concentration in the experimental samples was extrapolated from the standard curve. The GSH concentration was calculated and expressed as μmol of GSH/mg protein.

### Measurements of enzymes

The activity of SOD was determined by the method of Marklund and Marklund 1979 [22], using inhibition of pyrogallol autoxidation at pH 8. The specific activity of SOD is expressed as units per mg protein per minute. The activity of GPx was measured by the method of Paglia and Valentine [23]. GPx catalysis the oxidation of glutathione by Cumene hydroperoxide. In the presence of glutathione reductase (GR) and NADPH, the oxidized glutathione is immediately converted to the reduced form with a concomitant oxidation of NADPH to NADP. The decrease in absorbance is measured at 340 nm. GR catalyzes the reduction of glutathione in the presence of NADPH, which is oxidized to NADP. The decrease in absorbance is measured at 340 nm. The levels of GPx and GR were expressed as U/mg protein. The CAT activity was assayed by H2O2 consumption, following Aebi’s method [24] and modified by Pieper et al. (1995) [25].

### Protein estimation

Protein was estimated in the subcellular fractions by the method of Bradford (1976) using bovine serum albumin (BSA) as standard [26].

### Statistical analysis

All experiments were carried out at least in duplicate. The each group consisted of eight rats. One way analysis of variance (ANOVA) was performed and Tukey *post-hoc* test was used for multiple comparisons. Statistical analyses were performed using the InStat 3.0 program. The results are expressed as mean ± SEM. Differences of *p* < 0.05 were considered significant.

## Results

**Fig. 1:**
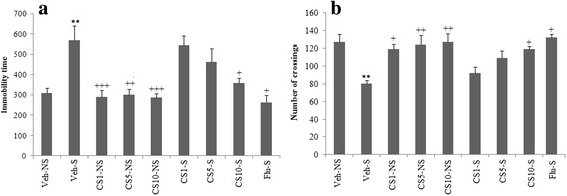
Effect of the treatment with CS and Flu (positive control) on immobility time in the FST (**a**) and on locomotor activity in the open field test (**b**) in the rat submitted to chronic restraint stress procedure. Each column represents the mean ± SEM (*n* = 8). Significantly different from Vthe eh-NS groups (**; *P* < 0.01). Significantly different from the Veh-S groups (+; *P* < 0.05, ++; *P* < 0.01, +++; *P* < 0.001)

**Fig. 2:**
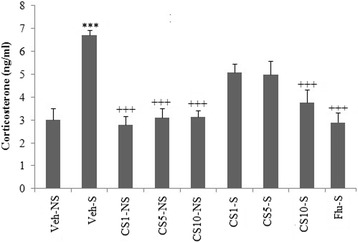
Effect of CS and Flu (positive control) on corticosterone levels in serum of immobilization stress and control groups (*n* = 8, for each group). Each measurement was done at least in triplicate and the values are the means ± SEM for eight rats in each group. Significantly different from the Veh-NS groups (***; *P* < 0.001). Significantly different from the Veh-S groups (+++; *P* < 0.001)

Results indicated that the rats submitted to the restraint stress (Veh-S) indicated an increase in The immobility time compared with the Veh-NS group (*p* < 0.01). However, the animals treated with CS (10 mg/kg) exhibited a significant decrease in the immobility time compared with the Veh-S group (*p* < 0.05) (Fig. 1a). Similar results were obtained with the10 mg/kg fluoxetine (Flu) administration in the stressed rats (Flu-S) (*p* < 0.05). Figure 1b indicates the evaluation of locomotor activity obtained in the open field test in the stressed or treated animals. This test revealed that stress decreased the number of crossing in rats submitted to the restraint stress versus the non-stress animals (*p* < 0.01). The treatment with CS (10 mg/kg) improved this alteration compared with the Veh-S group (*p* < 0.05). The administration of Flu (10 mg/kg) caused the similar results on the locomotor activity obtained in the open field test (*p* < 0.05). The serum corticosterone level of the Veh-S group was significantly higher than those of the Veh-NS (*P* < 0.001). The serum corticosterone level in the CS10-S group was significantly lower than those of the Veh-S group (*P* < 0.001). The Flu-S group acted as a positive control (Fig. 2). The brain MDA, GSH, SOD, GPx, GR, and CAT levels in all groups are shown in Table 1. The MDA level of the Veh-S group was significantly higher than those of the Veh-NS (*P* < 0.001). The MDA level and positive control (Flu-S) significantly decreased in the CS1-S, CS5-S and CS10-S groups compared to the Veh-S groups (*P* < 0.001, *P* < 0.01, *P* < 0.001, respectively).

**Table 1:** Effect of CS and Flu (positive control) on the MDA (nmol/mgp), GSH (μmol/mgp), SOD (U/mgp), GPx (U/mgp), GR (U/mgp), and CAT (U/mgp) levels in brain of immobilization stress and control groups (*n* = 8, for each group)

Brain	MDA	GSH	SOD	GPx	GR	CAT
Veh-NS	4.02 ± 0.74	6.13 ± 0.69	1.32 ± 0.09	0.69 ± 0.07	0.51 ± 0.05	3.42 ± 0.96
Veh-S	8.97 ± 0.88***	2.87 ± 0.71**	0.61 ± 0.11**	0.35 ± 0.10**	0.26 ± 0.02*	1.08 ± 0.21*
CS1-NS	4.12 ± 0.31++	5.89 ± 0.31+	1.01 ± 0.13	0.59 ± 0.10	0.50 ± 0.06+	3.00 ± 0.34
CS5-NS	3.89 ± 0.49+++	6.23 ± 0.29++	1.13 ± 0.10	0.63 ± 0.11+	0.48 ± 0.08+	3.12 ± 0.41+
CS10-NS	4.06 ± 0.97+++	6.04 ± 0.97++	1.28 ± 0.07++	0.71 ± 0.06++	0.53 ± 0.04++	3.47 ± 0.38++
CS1-S	7.88 ± 0.65*++	3.07 ± 0.55*	0.73 ± 0.15*	0.41 ± 0.05	0.32 ± 0.01	1.26 ± 0.25
CS5-S	6.43 ± 0.75***+++	4.12 ± 0.19	0.96 ± 0.20	0.52 ± 0.02	0.39 ± 0.03	1.77 ± 0.30
CS10-S	5.06 ± 1.08+++	5.67 ± 0.61+	1.17 ± 0.09+	0.61 ± 0.07	0.45 ± 0.07	3.10 ± 0.14+
Flu-S	4.66 ± 0.62+++	5.88 ± 0.73+	1.28 ± 0.19+	0.66 ± 0.10	0.49 ± 0.09	3.31 ± 0.39+

Each measurement was done at least in triplicate and the values are the means ± SEM for eight rats in each group

Significantly different from the Veh-NS groups (*; *P* < 0. 05, **; *P* < 0. 01, ***; *P* < 0.001)

Significantly different from the Veh-S groups (+; *P* < 0.05, ++; *P* < 0.05, +++; *P* < 0.001)

The GSH level of the Veh-S group was significantly lower than those of the Veh-NS group (*P* < 0.01). The GSH level in the CS10-S group was significantly higher than those of the Veh-S group (*P* < 0.05). The activities of SOD, GPx, GR, and CAT in the Veh-S group were significantly lower than those of the Veh-NS group (*P* < 0.01, *P* < 0.01, *P* < 0.05, *P* < 0.05, respectively). The SOD and CAT activities in the CS10-S groups were significantly higher than those of the Veh-S groups. The increases were obtained with the fluoxetine (Flu) (10 mg/kg) injection in the stressed rats (Flu-S) (*P* < 0.05).

**Fig. 3:**
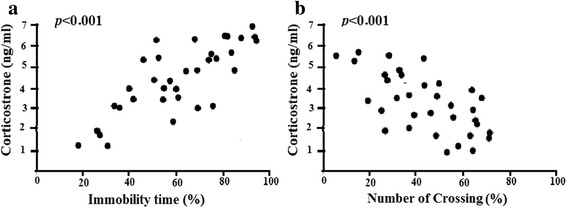
The behavioral results were correlated with relevant biological outcomes. Panel (**a**) shows the correlation between the immobility time in OFT and hippocampus corticosterone content. Panel (**b**) shows the correlation between the number of crossing in OFT and hippocampus corticosterone content

The correlations between the behavioral results and biological outcomes are shown in Fig. 3. The immobility time in the OFT was positively correlated with the serum corticosterone content (rp = 0.737, *P* < 0.001) (Fig. 3-a). The immobility time in the OFT was negatively correlated with the serum corticosterone content time (Fig. 3-b).

## Discussion

**Fig. 4:**
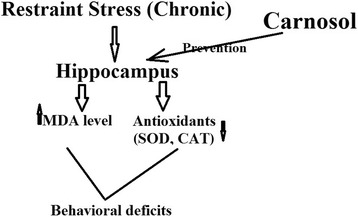
A graphical abstract

After 3 weeks of the chronic restraint stress (1 h/day), the rats showed more depression-like behavior (decrease in the immobility time in the FTS, and also decreased the number of crossing in the OFT). These behavioral changes were paralleled by biochemical alterations, including higher serum levels of corticosterone and lipid peroxidation marker (MDA) in the brain, that accompanied with the lower levels of GSH and antioxidant enzymes. The endocrine, oxidative, and nervous systems were induced to exert a chronic stress response in the body. The hypothalamic–pituitary–adrenocortical (HPA) activation is one of the main pathways in the stress response [27]. In the current study, the increase in serum corticosterone level was correlated with depression-like behavior in the FTS and OFT. The present results showed the modulatory effects of CS administration to improve the serum corticosterone level in the rats submitted to chronic stress. The chronic stress exposure can disturb the oxidant and antioxidant balance, and induce a large production of free radicals parallel to suppress antioxidant capacity. Similar to the previous studies, the present findings indicated that the levels of oxidative marker (MDA) increased after the chronic restraint stress, and the levels of antioxidant indices (including GSH, SOD, GPx, GR, and CAT) decreased [28, 29]. However, the CS treatment ameliorated these alterations in the rats submitted to chronic stress. It was also observed that the oxidative regulated activity of CS may be correlated with the serum corticosterone level. To the best of our knowledge, this is the first report showing that the CS treatment prevented behavioral alterations through modulating the oxidative response of the brain in the rats subjected to the chronic restraint stress procedure. The increased MDA as well as decreased antioxidant levels, confirmed the occurrence of oxidative damage in the hippocampus of rats submitted to the chronic stress. This alteration was also observed in the rodents submitted to chronic restraint stress by this procedure in several studies [30, 31]. Furthermore, the CS treatment during chronic restraint stress prevented the enhanced hippocampal lipid peroxidation, suggesting the occurrence of a neuro-protective effect in the rat. The oxidative changes likely are the main cause of brain damage in the pathophysiology of stress-induced depression. It was also reported that major depression and lipid peroxidation can be associated with human [32]. Indeed, the enzymatic and non-enzymatic antioxidants have been involved in preventing depressant conditions [33]. In line with our findings, the studies also found that the GSH alterations in the hippocampus of rats submitted to the chronic restraint stress as well as in depressive human serum [34]. The GSH related antioxidant system was also assessed by measuring the GPx and GR activities. Regarding these parameters, the decreased GPx and GR activities were found in the hippocampus of rats submitted to the chronic restraint stress procedure, a response that is probably related to an increase in the ROS production following the chronic stressor or decrease in the antioxidant production [35]. The overproduction of superoxide anion (O2•−) is one of the main factors involved in the stress-induced oxidative damage in the brain [36]. The present study has been indicated that the hippocampal activity of SOD was decreased in the rats submitted to chronic restraint stress. The increased ROS lowered the nuclear factor (erythroid-derived-2)-like 2 (Nrf-2), a primary transcriptional regulator of a majority of antioxidants, including SOD, GPx, GR, and CAT [16]. This may be a possible explanation for the decreased SOD, GPx, GR, and CAT activities that was seen after the chronic restraint stress. The stressed group treated with CS showed a higher increase in the activities of these enzymes as compared with the stressed rats treated with vehicle. Considering that, the neuroprotective effect of CS may be mediated by SOD, GPx, GR, and CAT, however, further studies are needed to determine the direct relationship between the antidepressant-like effect of CS and the antioxidant response. Despite the fact that major depression is one of the most common psychiatric disorders and is associated with high rates of disability, however, the therapeutic alternatives present has a variety of side effects and a long period of several weeks, in the onset of action. It has been shown that antioxidant compounds, including CS are considered as a new therapeutic option in the treatment of neuronal diseases. Since excitotoxicity and oxidative stress are found to act synergistically to induce neuronal damage [16], CS may be suggested as a putative antidepressant agent (Fig. 4).

## Conclusion

In conclusion, the present study showed that the CS administration prevented behavioral alterations via the modulating hippocampal oxidative response in the animals subjected to the chronic restraint stress procedure.
